# Editorial: Artificial intelligence and telemedicine: applications to vascular neurology, neuro-ophthalmology, neuro-otology, and epilepsy: advancing teleneurology with artificial intelligence and digital biomarkers

**DOI:** 10.3389/fneur.2025.1582142

**Published:** 2025-03-25

**Authors:** Jonathan D. Browne, Kemar E. Green

**Affiliations:** Department of Neurology, Johns Hopkins Medicine, Baltimore, MD, United States

**Keywords:** digital biomarkers, precision neurology, artificial intelligence, telemedicine, neuro-ophthalmology

Artificial intelligence (AI) continues to have an expanding role in clinical neurology. This Research Topic, *Artificial Intelligence and Telemedicine: applications to vascular neurology, neuro-ophthalmology, neuro-otology, and epilepsy* (2024–2025), explored emerging AI-driven technology (digital biomarker), particularly with pupillometry and eye movements. This research highlights the dynamic relationship between this novel technology and teleneurology.

## Artificial intelligence and pupillometry

Pupillometry is a valuable tool for characterizing neurological function, and two studies within this Research Topic examined AI-based pupillometry systems (Jaworski et al., Bogucki et al.). Jaworski et al. described a novel AI-driven mobile pupillometry system that demonstrated comparability to clinical-grade devices and identified significant correlations between pupillary responses and glaucoma-related markers. Conventional assessments of glaucoma progression often rely on subjective measures, whereas objective metrics that detect subtle changes in pupillary response can improve disease management. Beyond glaucoma, these techniques can be applied across various neurological domains to facilitate precision diagnostics, enable early intervention strategies, and assess treatment responses in both ophthalmic and neurologic disorders.

Building upon this, Bogucki et al. introduced a machine learning algorithm for correcting pupillary response parameters for variable ambient light conditions as well as the Pupil Reactivity (PuRe) score as an AI-derived metric to quantify pupillary response robustness. While devices such as smartphones are often convenient platforms, there can be limitations based on external conditions which this research shows can be mitigated (1). These developments are important when considering the accessibility of pupillometry in remote settings for making nuanced decisions about neurological conditions, such as traumatic brain injury, optic neuropathy, neurodegenerative disease, and more.

## Artificial intelligence and eye movements

Eye movements are another key area of neurological biomarkers (2), and AI has the potential to support critical medical decision-making (3, 4). Duvieusart et al. explored how AI-assisted eye movement analysis can improve diagnostic accuracy, finding that machine learning models trained on gaze test data were able to successfully classify acute vestibular syndrome (AVS) cases as either central (e.g., stroke-related) or peripheral (e.g., vestibular neuritis, benign paroxysmal positional vertical, etc.). As a common consultation from the emergency department to neurology, AVS evaluation must be timely and accurate (5). Further complicating the matter, in remote settings there may not be specialists present to make an accurate in-person assessment (1, 6, 7). AI-driven digital biomarkers continue to be refined and will bridge the diagnostic gap for non-specialist clinicians and support accurate triage in the emergency setting. This can be easily paired with telemedicine as a quick, cost-effective method to facilitate a collaborative diagnostic process.

Automated nystagmus tracking similarly is essential in neuro-vestibular and neuro-ophthalmology disorders (8–10). Cho et al. presented a deep learning model capable of detecting and tracking nystagmus in real time using object segmentation algorithms to isolate and analyze eye movements. This streamlined AI-driven approach achieved accuracy with object segmentation as well as predicting nystagmus direction. In a practical sense, this could help reduce reliance on unnecessary neuroimaging in the setting of clear peripheral vertigo conditions while facilitating prompt intervention. Tracking nystagmus patterns over time can also provide valuable insight into disease progression and treatment efficacy, as mentioned before for pupillometry. Brief clinical observations may not capture episodic eye movement patterns while AI-based tracking can be used for real-time long-term monitoring.

## The impact of artificial intelligence in teleneurology

This Research Topic highlights the expanding role of AI in augmenting teleneurology. AI-driven technologies are redefining conventional biomarkers—including pupillometry, ocular motor function, vestibular assessments, and retinal imaging—enhancing clinical practice and deepening our understanding of neurological diseases (3, 4). The four studies reviewed here highlight the growing role of AI in improving real-time diagnostics, streamlining clinical workflows, and expanding access to remote healthcare services (Jaworski et al., Bogucki et al., Duvieusart et al., Cho et al.).

While these studies focus mainly on ocular digital biomarkers, there is still ongoing potential for AI to expand in other areas of neurology such as stroke, epilepsy, neuromuscular disorders, movement disorders, etc. For example, AI shows utility with analysis of subtle abnormal human kinematics (e.g., limb movements or gait) as well as with interpreting complicated electrographic patterns on EEG. The synergistic potential of clinicians and researchers with this technology will enable better patient outcomes. Ongoing challenges include validation, regulatory and ethical considerations, and integration into clinical workflow. Continued interdisciplinary collaboration between neurologists, engineers, and AI specialists is crucial for realizing the potential of AI within neurology. There is a need for a unified seamless platform that can incorporate multi-modal tracking which can revolutionize detection and monitoring, especially considering the expanding spectrum of common and rare neurological diseases.
